# The Effect of a Multi-Strategy Program on Developing Social Behaviors Based on Pender’s Health Promotion Model to Prevent Loneliness of Old Women Referred to Gonabad Urban Health Centers

**Published:** 2015-04

**Authors:** Mehri Alaviani, Shahla Khosravan, Ali Alami, Mahdi Moshki

**Affiliations:** 1 Department of Community and Mental Health Nursing, School of Nursing and Midwifery, Maragheh Faculty of Medical Sciences, Maragheh, Iran;; 2 Department of Community and Mental Health, School of Nursing and Midwifery, Social Determinants of Health Research Centre, School of Nursing and Midwifery, Gonabad University of Medical Sciences, Gonabad, Iran;; 3 Department of Public Health, School of Health; Social Determinants of Health Research Center, Gonabad University of Medical Sciences, Gonabad, Iran;; 4 Department of Public Health, School of Health; Social Development & Health Promotion Research Center, Gonabad University of Medical Sciences, Gonabad, Iran

**Keywords:** Aging, Female, Loneliness, Model, Pender’s Health Promotion

## Abstract

**Background:**

Loneliness is one of the most significant problems during aging. This research has been done to determine the effect of a multi-strategy program based on Pender’s Health Promotion model to prevent loneliness of elderly women by improving social relationships.

**Methods:**

In this quasi-experimental study done in 2013 from January to November, 150 old women suffering medium loneliness referred to Gonabad urban Health Centers were enrolled. Data were gathered using Russell’s UCLA loneliness questionnaire and the questionnaires based on Pender’s Health Promotion Model about loneliness. The results were analyzed by descriptive statistics and Chi-square, T-pair, and independent-T tests through SPSS, version 20.

**Results:**

Loneliness decreased significantly in the interventional group compared to the control group (P<0.00). In addition, mean scores related to variables of Health Promotion Model (received benefits and barriers, self-efficacy, interpersonal effectives of loneliness) in both groups were significantly different before and after the study (P<0.05).

**Conclusion:**

Constructs of Pender’s Health Promotion Model can be used as a framework for planning interventions in order to anticipate, improve and modify related behaviors related to loneliness in old women.

## Introduction

According to World Health Organization definitions, aging occurs after 60 and causes physiological, mental and social changes.^1^ During recent years, the number of elderly people with increasing life expectancy has increased, so that one out of 10 people is more than 65 years old.^2^ According to a census which was conducted in 2011, 5.7% of Iran population are elderly and this rate is expected to increase to 19% in 2020.^3^ Therefore, we can say Iran in the current conditions is passing through young to elderly people and soon it will join to countries with the aging demographics.^4^ At the beginning of oldness, gradually people not only lose their physiologic performances but also their mental and socials ones, so suffering numerous problems such as loneliness.^4^^,^^5^ Loneliness does not mean living lonely; rather, it is felt when social interactions got damaged quantitatively or qualitatively.^6^ Since the elderly are faced with problems related to social interactions and interpersonal relations, many of them define aging period as a loneliness period and look at it as an unpleasant experience.^4^ Loneliness is a widespread phenomenon and near 25 to 50 percent of the total world population experiences it.^7^ This is more common among women.^8^ And it is an important factor in incidence, susceptibility or reinforcement of mental and physical diseases related to aging period, such as depression, suicide and severe disappointment, social isolation, impatience, anxiety, disturbance of self-care behaviors, and disruption in physical health problems such as disruption in immunity system function, nutrition and sleep.^6^^,^^8^ In order to help them cope with loneliness in elderly people, we can use numerous ways such as keeping animals,^9^ Gestalt Group Therapy,^10^ cognitive counseling with individual, music therapy, telling memories, training society in order to change individuals attitudes regarding aging period, and drug therapy.^11^^,^^12^ According to Robertsa and Pettigrewa’s research and based on interviews with the elderly, two general approaches are obtained to prevent and improve loneliness:

1. developing social behaviors (in group) by focusing on relationships with friends and family as an emotional source, and attendance in parties as a way to maintain social contacts
2. Focus on developing non-social behaviors (individually) by spending a definite time for activities like reading and gardening.^13^

Peplau and Perlman prescribe improving social behaviors to match with loneliness.^14^ Eric et.al in their study recommended using operations based on cognitive therapy and improving social relations in order to modify loneliness. They also concluded in comparing using these two methods- active methods of coping with loneliness (improving social relations) and inactive methods of coping with loneliness (reducing expectations about social relations and focusing on developing individual behaviors)- younger, healthy, educated and more confident elderly who had a job until midlife usually use these active matching methods.^15^

Experts believe that any attempts which lead to removing loneliness in the elderly is an obstacle against complicated mental problems of the elderly and successful treatment of loneliness may decrease the risk of serious complications such as depression.^11^ Due to constant contact with the elderly, many of health care providers, especially nurses, have a unique status in identifying and interfering loneliness.^3^ Studies have shown that in comparison with general interventions, interventions with individuals needs are more effective.^16^ Nurses use these interventions and different models as a performance guide in order to plann for changing behaviors and health promotion. One of these models is Pender’s Health Promotion Model and one of its features is intervention based on initial assessments.^17^ Pender provided the first version of health promotion model in 1982 and it is presented as a framework to assimilate nursing and behavioral science attitudes about effective factors on healthy behavior and as a guid for searching complicated mental-biological processes that stimulate individuals to be involved in reinforcing health behaviors.^18^ Health Promotion Model is based on social cognitive theory in which cognitive-perception factors (such as perceived benefits and barriers) have influence on involvement in health promotion behaviors and adjuster factors (like demographic factors, effective interpersonal factors, and behavioral factors) as a casual interaction factor affecting cognitive-perception process, thus indirectly determining healthy behaviors.^18^^,^^19^ This model emphasizes health promotion behaviors, recognizing behaviors and personal factors with increased efficacy and perception, reforming and strengthening behaviors and improving communication and situations. Therefore, this is a model which is potentially used during lifetime especially aging period when people are more vulnerable for various reasons.^18^ Nurses believe that identifying and providing applicable solutions in order to health improvement at mental- social domain of these people and in this regard reducing their loneliness based on these models are inevitable. So, this research was done to determine the effect of a multi- strategy program on developing social behaviors based on Pender’s Health Promotion model to prevent loneliness of old women referred to Gonabad urban Health Centers.

## Materials and Methods

This study was a quasi-experimental research  conducted in 2013 from January to November. The statistical population of this study was old women between 60-74 years who referred to Gonabad urban Health Centers of Gonabad, one of east cities of Iran, with a moderate loneliness level (35-48) through Russell’s questionnaire; they were identified in the first stage of this study.^20^ In this study, we used convenience sampling with Balanced Block Randomization to guarantee balance in numbers during intervention. To do this, 150 Persian speaking subjects who met the inclusion, criteria had appropriate speaking and listening ability, and signed informed consents were selected to participant in this study. We divided the subjects into the intervention and control groups. There was a total of 6 blocks with 4 patients in each block. Based on previous research (3), the estimated sample volume for this study was 120 for two groups as below:

n=2(Z1-α2+Z1-β)2∆2

By considering the attrition rate (20%) in research process, all of these subjects (150) who were city dwellers were included in the study. Exclusion criteria of this research were discontinuing participation, being absent for one session, experiencing grief during the intervention and follow up phases. These criteria led to elimination of 10 subjects from the intervention group due to absence in educational class or lack of participation in the post-test.  

Data gathering tools included Revised loneliness scale questionnaire of UCLA (University of California Los Angeles), and a six part researcher-made questionnaire according to the constructs of pander’s health promotion model. 

Revised loneliness scale questionnaire of UCLA was made by Russell et al. in 1980. This questionnaire consists of 20 questions and individuals’ score is gained through the sum of all 20 questions. The scoring method of this scale includes describing multiple choice phrases to be elected by individuals. These options are “never”, “rarely”, “sometimes”, and “often”. Scoring of the mentioned tool is in a way that some of its materials are scored reversely. The minimum score is 20 and maximum 80. Scores between 20-34 show slight loneliness equivalent with no loneliness, about 35-48 is medium loneliness and up to 48 is severe loneliness. Reliability is reported 0.89 through test- retest method by Russell.^21^ Also, Russell and Ferguson in 1998, in another test, have reported its reliability about 0.78.^22^ In Iran, Soudani et.al in a study reported stability of this scale 0.81 by using Cronbach’s alpha method.^23^

The researcher-made questionnaire was made up of five parts; the first part contained demographic characteristics with 7 questions, the second  part had 7 questions about “Perceived benefits” of loneliness (like I am not ok, the others’ trouble becomes less, I enjoy my life more than before, my grief diminishes), the third has perceived barriers including 14 questions (like I have no friends, my social relation is weak, I don’t trust on others, nobody is intimate with me, I don’t know myself as a member of my friends group), the forth part perceived self-efficacy including 6 questions( like I can communicate with others, I can take over my grief and sorrow, I can find many friends) and based on Likert scale each of them are graded through 4 options – from totally disagree (1 score) to totally agree (4 score), and the last part  was about  interpersonal influences including 5 questions (who is your supporter and provider for your attendance in friends gathering and communicating when you feel lonely?) which based on Likert scale it is graded into 4 options – not at all (0 score), to some extent (1 score), much (2 score) and very much (3score) and total score is calculated through the mean scores. In order to evaluate the questionnaire’s reliability, we used content validity methods. The questionnaire is prepared based on health promotion model and according to valid scientific resources and also its content validity was confirmed by 14 academic members. Assessing the questionnaire reliability was done using Cronbach’s alpha test. The total internal correlation of health promotion models questionnaire was 79.5% (respectively perceived benefits and barriers were 80 and 82%, perceived self efficacy 79%, and interpersonal influences 82%).

After choosing the subjects, at first a pre-test was conducted for all of them using the researcher made questionnaire; and then we analyzed these questionnaires to plan a multi-strategy program based on constructs Pender’s Health Promotion model (Perceived benefits, barriers, self- efficacy, interpersonal influences, and behaviors) with the aim of reduction of loneliness in the elderly women by improving social relationships and efficacy.^24^

Then, the subjects were blocked by random assignment method and divided into two intervention and control groups. In order to provide better intervention, the intervention group was divided into 3 sub-groups of 25 subjects. And four sessions (two times in a week), each of which lasting for 60 minutes, were held by monitoring the first author in the conference room of health centers. In this intervention and in the first session, by using lecture and question-answer methods, the two first authors talked about the definitions, causes, clinical symptoms and complication of loneliness. And at the end, a summary of all contents was given to them and they were asked to think about this question for the next session (when you feel lonely, what do you do?). The goal of the second session was empowerment of subjects to improve social relationship as an important factor to reduce loneliness. The section was started by a short review on related contents of the previous session and discussion about the activities and behaviors when feeling lonely. Then Effective Interpersonal interaction (like realization of expectation of interpersonal interaction with family, friends, and neighbors, effective techniques to improve interactions with social network, trust in others, group formation to help others) were explained to them. Finally, they considered special practical practices to motivate and create self-efficacy in subjects with improving social interaction (such as group walking, participating in Quran meetings, visiting children) and they were asked to perform it until the next session. In the third and fourth sessions, their achievements were checked and if they achieved their goal, they were faced with positive feedback and the researcher reviewed the benefits of practical practices. Then, if they did not do their assignment, the barriers would be checked and the reprogramming deviation would be refined. At the end of the fourth session, for their familiarity with public places and improving social interactions, the participants went to nursing home. Between these sessions, the first author visited the subjects in their house in order to observe group works and participant behavior changes. A few days later, to increase self-esteem and positive self-evaluation, by comparing themselves to someone better or worse, the subjects visited an elderly nursing home and a Children Charity Organization. Finally, after one month from the last intervention, again research tools were completed by the intervention and control samples and their scores were considered as a post-test. Then, the gathered data were analyzed in SPSS, version 20, after assessing normality by P-P PLOT and Q-Q PLOT. In order to describe characteristics of research units, we used descriptive statistics like frequency distribution, mean (standard deviation) and also analytic statistical tests such as Chi-Square, T-paired, and T-independent.

The Institutional Review Board and Ethics Committee of Gonabad University of Medical Sciences approved the study. Also, we explained the aim and process of the study to the study participants and guaranteed the confidentiality of their personal information. We also ensured them that both participation in and withdrawal from the study were voluntary. Finally, a written informed consent was obtained from the entire study participants. 

## Results

The results of demographic characteristics of the two intervention and control groups are shown in Table 1. According the Table, before the intervention, there were not any significant differences between the two groups in terms of their marital status, educational level, occupation, child number, family status, earnings and chronic disease (Table 1).

**Table 1:** comparison of demographic variables between intervention and control groups

**Socio-demographic ** **characteristics**	**Intervention**		**Control**		**Tests and P value**
	**n**	**%**	**n**	**%**	
Marital Status					0.47
Married	54	72	50	66.6	
Single	21	28	25	33.4	Chi-Square
Educational level					0.86
Illiterate	28	37.3	27	36	
Literate	47	62.7	48	64	Chi-Square
Occupation					0.36
Retired	4	3.5	1	1.3	
House keeper	71	94.7	74	98.7	Fisher’s Exact Test
Status					0.53
Living alone	17	22.7	15	20	
Living with wife	37	49.3	30	40	
Living with wife and children	15	20	21	28	
Living with children					
Status	6	8	8	10.7	Chi-Square
Other	0	0	1	1.3	
The level of income					0.44
Sufficient	20	26.7	16	21.3	Chi-Square
Insufficient	55	73.3	59	78.7	
Chronic disease status					0.55
Having chronic disease	60	80	57	76	Chi-Square
Not having chronic disease	15	20	18	24	

Loneliness scores are shown in Table 2. As shown in this table, the average score of loneliness in the intervention group showed a significant difference, in comparison with the control group after the study (P<0.001).

**Table 2:** Comparison of mean (standard deviation) score of feeling loneliness in the intervention and control groups before and after study

**Loneliness**	**Group (mean±SD )**		**P value** **Independent samples t-test**
	**Intervention**	**Control**	
Before study	43.4±3.7	42.5±3.9	0.151 P<0.001
After study	30.5±4.2	42.7±3.8	
P value paired samples t-test	P<0.001	P=0.2	

Results of this study indicated that before the intervention there was no significant difference in the mean score of health promotion model constructs perceived barriers, benefits, self-efficacy, and interpersonal influences in both groups. However, after the intervention between the two groups in terms of the above variables, significant differences were observed (P<0.001). In the intervention group, the mean score of perceived benefits of loneliness (social isolation) was decreased, while the mean score of barriers to stop loneliness (to increase social contacts), perceived social self-efficacy and interpersonal influences increased (Table 3).

**Table 3:** Comparison of mean (standard deviation) Pender’s health promotion model constructs related to loneliness in the intervention and control groups before and after the intervention

**The score of health promotion model constructs’**		**mean±SD**		**P value** **Independent samples t-test**
		**Intervention**	**Control**	
Perceived benefits	Before	13.3±3.0	13.6±2.8	0.600
	After	9.5±1.7	14.0±2.6	P<0.001
Perceived barriers	Before	42.7±6.2	41.3±7.3	0.198
	After	27.8±5.5	41.6±6.3	P<0.001
Perceived self-efficacy	Before	11.0±2.5	11.2±2.4	0.582
	After	18.0±3.1	11.1±2.5	P<0.001
Interpersonal influences	Before	3.5±1.4	3.5±1.4	0.955
	After	9.0±2.2	3.5±1.9	P<0.001

Interpersonal influences (supportive relationship), which were effective in reducing loneliness, were improved in the intervention group (Table 4).

**Table 4:** Comparison of interpersonal influences (relations) before and after the intervention in the intervention and control groups

**Interpersonal influences**	**Before intervention**				*P value					*P value
	**Intervention**		**Control**			**Intervention**		**Control**		
	**Yes**	**No**	**Yes**	**No**		**Yes**	**No**	**Yes**	**No**	
Family	46 (61.3%)	29 (38.7%)	48 (64%)	27 (36%)	0.736	51 (78.5%)	14 (21.5)	43 (57.3%)	32 (42.7%)	0.008
Friends	30 (40%)	45 (60%)	26 (34.7%)	49 (65.3%)	0.500	54 (83.1%)	11 (16.9%)	38 (50.7%)	37 (49.3%)	P<0.001
Neighbors	59 (78.7%)	16 (21.3%)	63 (84%)	12 (16%)	P=0.402	60 (92.3%)	5 (7.7%)	50 (66.7%)	25 (33.3%)	P<0.001
Cares and health providers	18 (24%)	57 (86%)	14 (18.7%)	14 (18.7%)	0.425	50 (76.9%)	15 (23.1%)	16 (21.3%)	59 (78.7%)	P<0.001

*Chi square Test

## Discussion

The aim of this intervention is to study the effect of designed intervention based on Pender’s Health Promotion Model in order to improve social relations in old women with loneliness. The results showed that the intervention reduced loneliness in the intervention group subjects. Although, according to authors’ search, there was no similar study about the effect of intervention based on this model in the elderly women who suffered loneliness, in line with the results of this study, interventions such as telling memories and music therapy lad to reduction of loneliness in the elderly people; one of its probable reasons is attending in groups, stabilizing social connections, and extending social networks^25^ which are the aim of the program and intervention in this study, as well. Previous studies showed that participating in prevention programs of social isolation including group discussion sessions and sharing experiences among the elderly people, and historical and famous places of city led to reduction of loneliness, depression and mental health of the elderly people.^26^ And it is similar to our results, too. Artnez et.al in their study and in line with our results showed that participating in active social programs like more responsibilities in house chores and encouraging to attend in social activities led to reduction of loneliness of the elderly in nursing home who are between 51-89 years old and recently became widow.^27^ A friendship enrichment program that was designed to improve friendship and self-esteem of older women based on feminist therapy, counseling re-evaluation, and self-help method, was successful to reduced loneliness in the intervention group.^28^ Although, similar to our study, these interventions decreased loneliness in the old women, this research was a short term multi-strategy program designed according to the participants’ needs, and it seems that it is more matched with our culture. For example, improving family relations and enhancing meaningful activities to promote social contact and spending time alone, which was suggested in Martina and Stevens’ study as a complementary intervention to increase the effect of friendship enrichment program, were consider in this study.

In addition, the results of this study indicated the scores of perceived barriers and benefits were decreased in the intervention group, while the perceived self-efficacy score was increased in this group. The results of other research were similar to the results of this study in that self-efficacy is one of the mental health dimensions the improvement of which will enhance people’s health promotion behavior.^29^^-^^32^

According the results of this study, the mean scores of interpersonal influences and frequency of social relations showed a significant difference with the control group. According to other studies, greater perceived availability of social support and higher levels of social network relationships were significantly related to a lower level of loneliness;^33^ such interventions as increasing understanding about receiving social supports, improving social behaviors, improving interpersonal relations through changing lifestyle and unfavorable behaviors will lead to reduction of loneliness.^32^^,^^34^^-^^37^

This study had a short  follow-up time  and also all of Pender’s constructs of health promotion model were not assessed. 

## Conclusion

The results of this study show that constructs of Pender’s health promotion nursing model can be used as a framework for planning interventions in order to anticipate, improve and modify related behaviors in loneliness. Researchers have recommended application of this model to improve other situations in the mental- social domain, especially by community nurses. 
